# Characterization of an *Enterococcus* sp. SMC-9 strain isolated from bile of a patient with cholangitis

**DOI:** 10.1371/journal.pone.0312953

**Published:** 2024-12-02

**Authors:** SooHo Yu, Minhee Kang, Yeonjae Yoo, Tae Yeul Kim, Hee Jae Huh, Nam Yong Lee

**Affiliations:** 1 Department of Laboratory Medicine and Genetics, Samsung Medical Center, Sungkyunkwan University School of Medicine, Seoul, South Korea; 2 Biomedical Engineering Research Center, Smart Healthcare Research Institute, Samsung Medical Center, Seoul, South Korea; 3 Department of Medical Device Management and Research, Samsung Advanced Institute for Health Sciences & Technology, Sungkyunkwan University, Seoul, South Korea; 4 Division of Environmental Science and Ecological Engineering, College of Life Science and Biotechnology, Korea University, Seoul, South Korea; Chandigarh University, INDIA

## Abstract

The genus *Enterococcus* is increasingly recognized for its involvement in various human infections, with several species known to be pathogenic. This study characterized *Enterococcus* sp. SMC-9, isolated from bile of a patient with cholangitis, and compared its characteristics with those of *Enterococcus montenegrensis* CoE-012-22^T^, recently isolated from dried beef sausage. A comprehensive analysis, encompassing phylogenetic, genomic, and phenotypic studies, confirmed that strain SMC-9 belongs to the same species as *E*. *montenegrensis* CoE-012-22^T^. However, comparative genomic analysis revealed key differences in virulence and antibiotic resistance gene profiles between the two strains. Notably, genes related to exopolysaccharide biosynthesis and the L-rhamnose biosynthesis pathway were found exclusively in strain SMC-9, suggesting their role in the strain’s colonization of the biliary tract and its involvement in cholangitis. Additionally, the tetracycline resistance gene *tet(M)*, which was absent in *E*. *montenegrensis* CoE-012-22^T^, was identified in strain SMC-9, explaining its high tetracycline minimum inhibitory concentration (>16 μg/mL). These findings highlight the unique pathogenic traits of strain SMC-9 compared to *E*. *montenegrensis* CoE-012-22^T^. Our study underscores the significant genetic and phenotypic variations that can exist among strains within the same species, highlighting the critical need for strain typing to assess their potential impact on patient outcomes and public health.

## Introduction

The genus *Enterococcus* was first described by Schleifer and Kilpper-Bälz [1] and currently comprises 62 species with a validly published and correct name [2]. Members of the genus *Enterococcus*, collectively termed enterococci, are Gram-stain-positive, catalase- and oxidase-negative, non-spore-forming, facultative anaerobic bacteria that produce lactic acid as the major product of carbohydrate fermentation. Enterococci are widely distributed in the environment, especially in soil, water, and plants, and are normal inhabitants of the gastrointestinal tract of humans and animals [3]. Enterococci have traditionally been considered to be of low pathogenicity but have recently emerged as a major cause of nosocomial infections worldwide [4]. To date, 17 enterococcal species have been associated with human infections, including bacteremia, endocarditis, catheter-related urinary tract infections, neonatal sepsis, surgical and burn wound infections, meningitis, and intra-abdominal and pelvic infections [5, 6]. Notably, enterococci are leading causative agents of biliary tract infections, with *Enterococcus faecalis* and *Enterococcus faecium* being the most commonly involved species.

The initial objective of this study was to investigate the characteristics and taxonomic status of strain SMC-9, isolated from bile of a patient with cholangitis in April 2021. At the time of isolation, strain SMC-9 was believed to represent a novel species within the genus *Enterococcus*. However, during the course of this research, a similar strain (CoE-012-22^T^) was isolated from dried beef sausage. Based on its phenotypic and genomic characteristics, this strain was considered to represent a novel species, for which the name *Enterococcus montenegrensis* sp. nov. is proposed. Additionally, strain CoE-012-22^T^ contains genes linked to hydrolase activity on ester bonds, carbohydrate transmembrane transporter activity, and tagatose-bisphosphate aldolase activity, which are known to improve food flavors or textures. Other genes identified are related to sialic acid lyase activity, DNA-binding transcription factor activity, flavin mononucleotide binding, and histidine-containing phosphotransfer, which play roles in signaling, immunity, and gene expression regulation [7]. Despite these findings, our study remains focused on the detailed characterization of strain SMC-9, as it was isolated from the bile of a cholangitis patient, unlike strain CoE-012-22^T^, which was isolated from food. We aimed to elucidate the unique pathogenic traits of strain SMC-9 that distinguish it from strain CoE-012-22^T^.

## Materials and methods

### Ethics statement

The present study was reviewed and approved by the Institutional Review Board of Samsung Medical Center, Seoul, South Korea (approval number: 2023-08-008). Given the retrospective study design, the requirement for informed consent was waived. A chart review was conducted from October to November 2023. No patient-identifying information was recorded.

### Strain isolation and patient history

Strain SMC-9 was isolated from the bile of a patient with cholangitis who had undergone percutaneous transhepatic biliary drainage (PTBD) due to malignant biliary obstruction. The patient presented to the emergency room with a high fever (40.1°C) and chills. Blood tests revealed anemia (hemoglobin: 8.6 g/dL), leukocytosis with neutrophilia (white blood cells: 12.7 × 10^9^/L; neutrophils: 96.1%), and elevated C-reactive protein (11.7 mg/dL). Liver function tests indicated obstructive jaundice, with total bilirubin of 3.8 mg/dL, direct bilirubin of 3.3 mg/dL, alkaline phosphatase of 1,296 U/L, and gamma-glutamyl transferase of 239 U/L. Suspecting acute cholangitis, bile and blood cultures were performed, and empirical antibiotic therapy (intravenous piperacillin/tazobactam 4.5 g every 8 hours) was initiated. A bile specimen collected through the PTBD tube was inoculated onto a blood agar plate and incubated aerobically at 37°C for 24 hours, resulting in the growth of cream-colored colonies. Initially, these colonies were identified as *Streptococcus bovis* using matrix-assisted laser desorption/ionization time-of-flight mass spectrometry (VITEK MS; bioMérieux, Marcy-l’Étoile, France) with a confidence value of 68.0%. However, further testing using an automated system for biochemical identification and antimicrobial susceptibility testing (AST) (VITEK 2; bioMérieux) identified the isolate, designated as SMC-9, as *E*. *faecium* with 99% probability. Empirical antibiotic therapy was continued for five days with a good clinical response; however, bile cultures persistently grew the same organism, and C-reactive protein levels remained elevated. The antibiotic regimen was then switched to intravenous tigecycline 50 mg every 12 hours. After eight days of tigecycline treatment, bile cultures turned negative, and the patient was referred to a palliative care hospital for supportive care.

### Antimicrobial susceptibility testing

AST was carried out using VITEK 2, with minimum inhibitory concentrations (MICs) interpreted based on the breakpoints described in the Clinical and Laboratory Standards Institute M100-Ed31 document [8].

### Phenotypic characterization

The cell morphology of strain SMC-9 was examined using transmission electron microscopy (TEM) (HT7700; Hitachi, Tokyo, Japan) at an accelerating voltage of 100 keV. Prior to analysis, the strain was cultured aerobically on MRS agar (Thermo Fisher Scientific, Waltham, MA, USA) at 37°C for 48 h. Sample preparation for TEM analysis was performed as previously described with minor modifications [9]. Briefly, bacterial cells were fixed with 2.5% glutaraldehyde and post-fixed with 2% osmium tetroxide. After dehydration in a graded series of ethanol, the cells were embedded in epoxy resin and sliced using an ultramicrotome (Leica EM UC7 Ultramicrotome; Leica Microsystems, Wetzlar, Germany). The ultrathin sections were then stained with uranyl acetate and lead citrate and examined using TEM.

Comparative phenotypic analysis of strain SMC-9 and closely related type strains was performed. The type strains used for comparison with strain SMC-9 were obtained from the National Collection of Industrial, Food and Marine Bacteria (*E*. *montenegrensis* CoE-012-22^T^ = NCIMB 15468^T^), the Japan Collection of Microorganisms (*Enterococcus saigonensis* JCM 31193^T^), the Korean Collection for Type Cultures (*Enterococcus canintestini* KCTC 21021^T^, *Enterococcus dispar* KCTC 13288^T^, and *Enterococcus asini* KCTC 13286^T^), and the Korean Agricultural Culture Collection (*Enterococcus diestrammenae* KACC 16708^T^). Catalase and oxidase activities were examined with 3% (v/v) hydrogen peroxide solution (Sigma‐Aldrich, St. Louis, MO, USA) and 1% (w/v) tetramethyl-p-phenylenediamine solution (bioMérieux), respectively. The presence of Lancefield group D antigen was tested using the Oxoid Streptococcal Grouping Kit (Thermo Fisher Scientific). Tolerance to bile and the ability to hydrolyze esculin were assessed on bile esculin agar (Merck, Darmstadt, Germany). Other biochemical characteristics were determined using API 20 Strep (bioMérieux).

The growth of strain SMC-9 and *E*. *montenegrensis* CoE-012-22^T^ under different NaCl concentrations, pH levels, and temperatures was tested. The salinity test was conducted on MRS agar with NaCl concentrations ranging from 0% to 8%, increasing in increments of 0.5%. The pH test was conducted at the optimal NaCl concentration determined earlier, with a pH range from 4.5 to 10.0 in intervals of 0.5. The growth was further investigated at different temperatures–4, 10, 15, 20, 25, 28, 30, 37, 40, 42, 45, and 50°C–under the optimal NaCl concentration and pH level.

### Whole-genome sequence analysis

Genomic DNA was extracted using the MG Genomic DNA Purification Kit (Macrogen, Seoul, South Korea), and sequencing libraries were prepared using the TruSeq DNA Nano Library Prep Kit (Illumina, San Diego, CA, USA). Whole-genome sequencing was performed using the HiSeq X Ten system (Illumina). Raw paired-end reads underwent trimming to remove adapter sequences, low-quality reads, and PhiX sequences using BBDuk v38.84 [10] Subsequently, the trimmed reads were de novo assembled using SPAdes v3.13.0 [11]. The assembled contigs were annotated using Prokka v1.14.6 [12] and the Rapid Annotation using Subsystem Technology (RAST) server v2.0 [13–15]. The comparison of gene functions between strain SMC-9 and strain CoE-012-22^T^ was performed using the RAST server v2.0 and the Kyoto Encyclopedia of Genes and Genomes (KEGG) v110.1 [16]. Pathogenicity potential was assessed using PathogenFinder v1.1 (database version: 2014) [17], and virulence genes were identified using VirulenceFinder v2.0.5 (database version: 2022-12-02) [18]. Antibiotic resistance genes were detected using the Comprehensive Antibiotic Resistance Database (CARD) v3.2.9 (database version: 2024-02-13) [19] and ResFinder v4.4.2 (database version: 2023-04-12) [20]. The DNA G+C content was calculated from the genome sequences. The circular genome was visualized using Proksee v1.0.0 [21].

### Phylogenetic and phylogenomic analyses

The full-length sequence of the 16S rRNA gene was retrieved from the genome sequence of strain SMC-9 and compared with the corresponding sequences of related strains within the genus *Enterococcus* available in the NCBI database (https://www.ncbi.nlm.nih.gov/genbank/). Sequence similarity was calculated using the pairwise sequence alignment tool in NCBI. Multiple sequence alignment was performed using Clustal X [22], and the phylogenetic tree was constructed using the neighbor-joining method with 1,000 bootstrap replicates in MEGA 11 software [23].

For phylogenomic analysis, genome sequences of related strains within the genus *Enterococcus* were downloaded from the RefSeq database (https://www.ncbi.nlm.nih.gov/refseq/). Average nucleotide identity (ANI) and *in silico* DNA-DNA hybridization (isDDH) values between strain SMC-9 and these strains were computed using the ANI calculator based on the OrthoANIu algorithm [24, 25] and the Genome-to-Genome Distance Calculator 3.0 with formula 2 [26, 27], respectively. A heatmap for strain SMC-9 and related enterococcal strains was generated based on OrthoANI values calculated using the OAT software [25]. Additionally, a phylogenomic tree was constructed using their genome sequences. Briefly, single-copy orthologs were identified using OrthoFinder v2.4.0 with the inflation parameter set to 3.0 [28]. The amino acid sequences of these orthologs were aligned using MAFFT v7.475 with the “—auto” option [29] and trimmed using Gblocks v0.91b [30]. The trimmed alignment was submitted to IQ-TREE v1.3.11.1 [31], where a maximum-likelihood tree was built using the LG substitution model selected by ModelFinder [32] and 100 bootstrap replicates. Type (Strain) Genome Server (https://tygs.dsmz.de) results, including species cluster, subspecies cluster, G+C content, delta statistics, genome size, and protein count, were incorporated alongside the phylogenomic tree [33].

## Results and discussion

### Phenotypic characteristics

**Fig 1:**
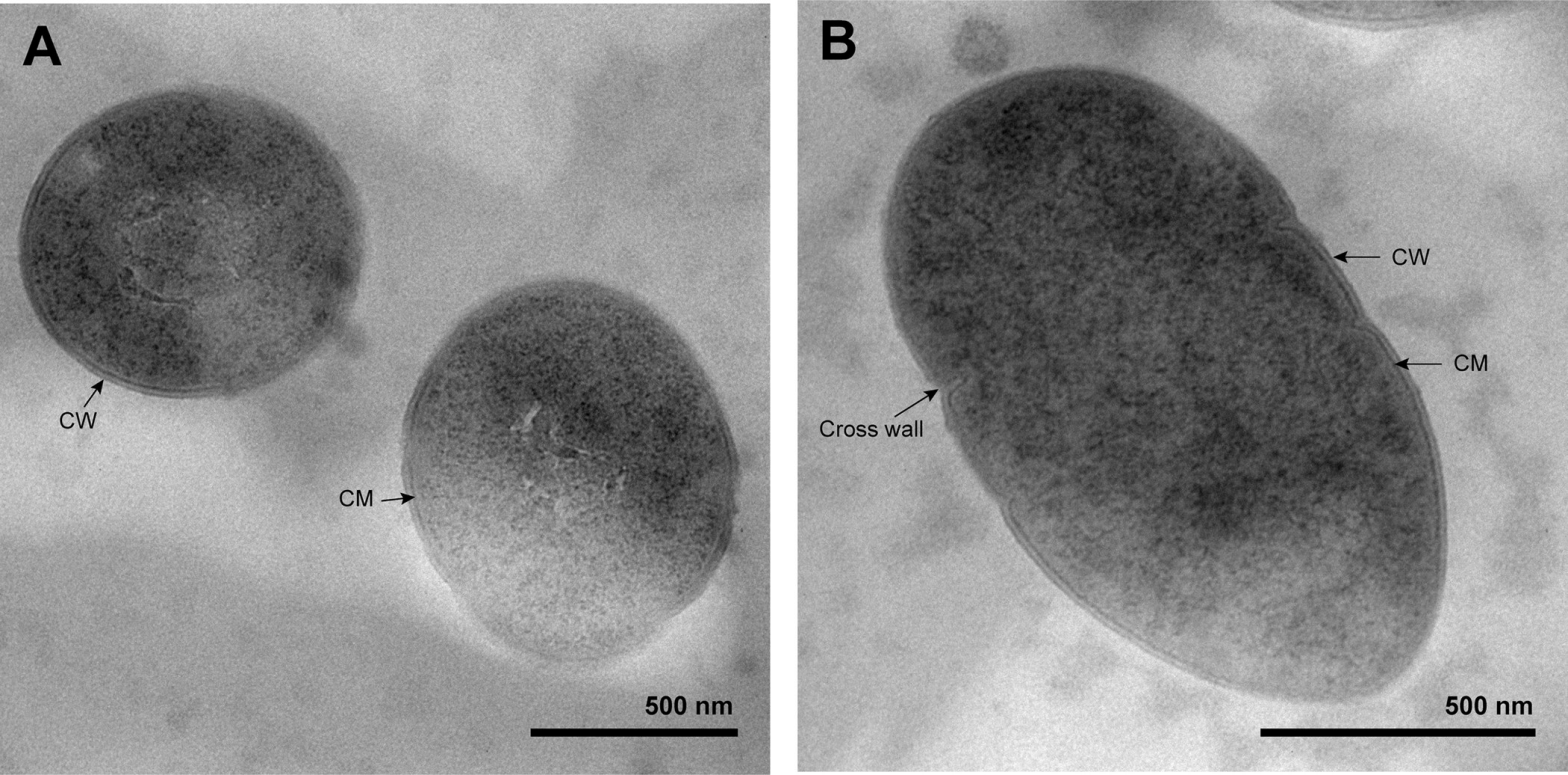
Transmission electron microscopy image of strain SMC-9 grown on MRS agar at 37°C for 48 h. The organism is spherical (A) or ovoid (B) in shape with 0.7–1.2 μm in diameter. The scale bar indicates 500 nm. CW, cell wall; CM, cytoplasmic membrane.

Colonies grown on MRS agar after incubation at 37°C for 48 h were circular, convex, entire, smooth, shiny, greyish-white, and 1–2 mm in diameter. Under TEM observation, the cells of strain SMC-9 were spherical or ovoid in shape with 0.7–1.2 μm in diameter. Flagella were not seen in the cells, suggesting that this strain is non-motile (Fig 1).

Strain SMC-9 exhibited distinct growth patterns compared to *E*. *montenegrensis* CoE-012-22^T^ in terms of salinity, pH, and temperature tolerance. The salinity tests showed that strain SMC-9 could grow in NaCl concentrations ranging from 0% to 5.5%, with optimal growth observed at a 3.0% NaCl concentration. In contrast, *E*. *montenegrensis* CoE-012-22^T^ could grow in NaCl concentrations ranging from 0% to 6.0%. When tested at a 3.0% NaCl concentration, strain SMC-9 exhibited growth between pH 5.0 and 7.5, with optimal growth observed within the pH range of 6.0 to 6.5. In comparison, *E*. *montenegrensis* CoE-012-22^T^ showed growth over a broader pH range, from 5.0 to 8.0. Under conditions of 3.0% NaCl and pH 6.5, *E*. *montenegrensis* CoE-012-22^T^ demonstrated growth across a wide temperature range of 4 to 50°C, while strain SMC-9 exhibited growth only within a narrower range of 25 to 37°C.

Strain SMC-9 lacked the Lancefield group D antigen and exhibited negative reactions in both catalase and oxidase tests. Despite being considered the same species, strain SMC-9 and *E*. *montenegrensis* CoE-012-22^T^ exhibited notable phenotypic differences. Specifically, strain SMC-9 was positive for hippuric acid hydrolysis, a trait not observed in *E*. *montenegrensis* CoE-012-22^T^. Conversely, *E*. *montenegrensis* CoE-012-22^T^ displayed α-galactosidase activity, which was absent in strain SMC-9. Furthermore, strain SMC-9 did not ferment D-raffinose and starch, both of which were acidified by *E*. *montenegrensis* CoE-012-22^T^ (Table 1).

**Table 1:** Differential phenotypic characteristics of strain SMC-9 and related type strains of the genus *Enterococcus*.

Characteristics	1	2	3	4	5	6	7
Growth at							
10°C	-	+	+	-	+	+	-
45°C	-	+	+	+	-	-	+
Group D antigen	-	N/A	-	-	-	-	+
API 20 Strep results:							
Acetoin production	+	+	+	+	+	-	-
Hippuric acid hydrolysis	+	-	-	-	-	-	-
β-glucosidase hydrolysis	+	+	+	+	+	+	+
Pyrrolidonyl arylamidase	+	+	+	+	-	-	+
α-galactosidase	-	+	+	+	-	-	-
β-glucuronidase	-	-	-	-	-	-	-
β-galactosidase	-	-	-	+	-	-	-
Alkaline phosphatase	-	-	-	-	-	-	-
Leucine aminopeptidase	+	+	+	+	-	-	-
Arginine dihydrolase	+	+	+	+	+	-	-
Acidification of							
D-ribose	+	+	+	+	+	-	-
L-arabinose	-	-	-	+	-	-	-
D-mannitol	-	-	-	-	-	-	-
D-sorbitol	-	-	-	-	-	-	-
D-lactose	+	+	+	+	+	+	-
D-trehalose	+	+	+	+	+	-	-
Inulin	-	-	-	-	-	-	-
D-raffinose	-	+	-	+	-	-	-
Starch	-	+	-	+	-	-	-
Glycogen	-	-	-	-	-	-	-

Strains: 1, SMC-9; 2, *E*. *montenegrensis* CoE-012-22^T^; 3, *E*. *canintestini* KCTC 21021^T^; 4, *E*. *saigonensis* JCM 31193^T^; 5, *E*. *dispar* KCTC 13288^T^; 6, *E*. *diestrammenae* KACC 16708^T^; 7, *E*. *asini* KCTC 13286^T^.

All data were obtained from the current study. +, Positive; -, Negative; N/A, not analyzed.

### 16S rRNA gene sequence and phylogenetic analysis

**Fig 2:**
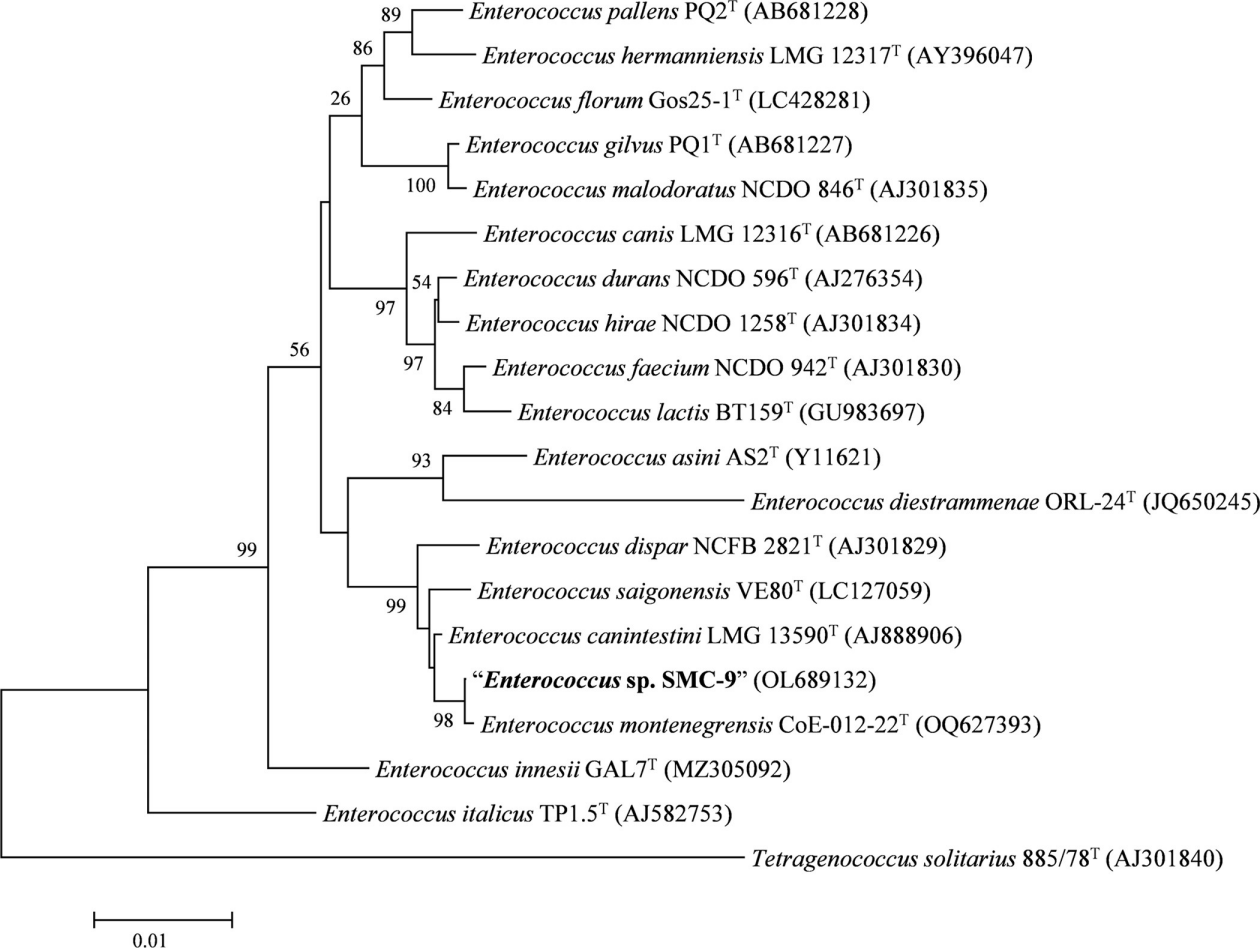
Neighbor-joining phylogenetic tree based on 16S rRNA gene sequences showing the relationships between strain SMC-9 and related type strains in the genus *Enterococcus*. Bootstrap values (≥50%) based on 1,000 replications are given at branch nodes. *Tetragenococcus solitarius* 885/78^T^ was used as an outgroup. Bar, 0.01 substitutions per nucleotide.

The full-length sequence (1,558 bp) of the 16S rRNA gene (GenBank accession number OL689132) was obtained from the whole genome sequence of strain SMC-9. Based on the 16S rRNA gene sequence, strain SMC-9 was most closely related to *E*. *montenegrensis* CoE-012-22^T^ (100%), followed by *E*. *canintestini* DSM 21207^T^ (99.6%), *E*. *saigonensis* VE80^T^ (99.6%) and *E*. *dispar* ATCC 51266^T^ (99.3%). In the phylogenetic tree based on 16S rRNA gene sequences (Fig 2), strain SMC-9 clustered closest with *E*. *montenegrensis* CoE-012-22^T^. Additionally, the strain formed a closely related clade with *E*. *canintestini* LMC 13590^T^, *E*. *saigonensis* VE80^T^, and *E*. *dispar* NCFB 2821^T^. The clustering observed in the phylogenetic tree is consistent with the numerical sequence similarities, providing support for the classification of strain SMC-9 as the most closely related to *E*. *montenegrensis* CoE-012-22^T^.

### Phylogenomic analysis and genomic characteristics

The genome size of strain SMC-9 was 2.81 Mb with a DNA G+C content of 37.5 mol%, which is within the range reported for *Enterococcus* species (genome size: 2.3 to 5.3 Mb; G+C content: 34 to 45 mol%) [34]. Genome annotation using Prokka predicted 2,639 coding sequences (CDS), 53 tRNA genes, 3 rRNA genes, and 1 tmRNA gene. The RAST server annotation predicted 2,713 CDS, of which 1,290 (47.5%) were classified into 353 subsystems. The most abundant subsystem category was carbohydrates (390 CDS), followed by amino acids and derivatives (273 CDS), protein metabolism (222 CDS), cell wall and capsule (133 CDS), and DNA metabolism (123 CDS) (S1 Fig). The circular genome map of strain SMC-9 is shown in S2 Fig.

**Fig 3:**
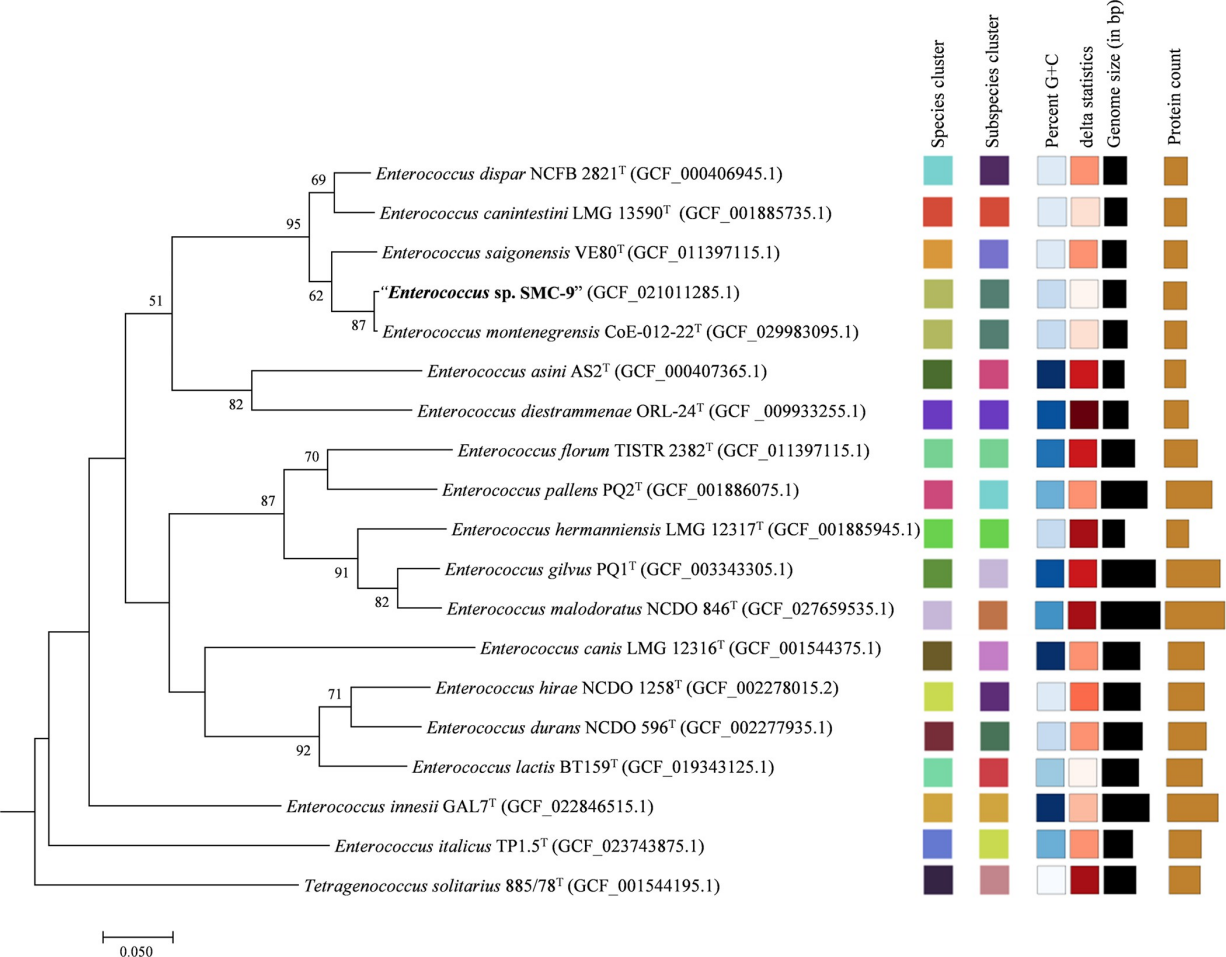
Phylogenomic tree of strain SMC-9 and related strains of the genus *Enterococcus*. The tree was reconstructed based on the 562 single-copy orthologue protein sequences. Bootstrap values (≥50%) based on 100 replications are given at branch nodes. Type Strain Genome Server analysis (TYGS) results, including species cluster, subspecies cluster, GC content, delta statistics, genome size, and protein count, were incorporated alongside the phylogenomic tree. *Tetragenococcus solitarius* 885/78^T^ was used as an outgroup.

**Fig 4:**
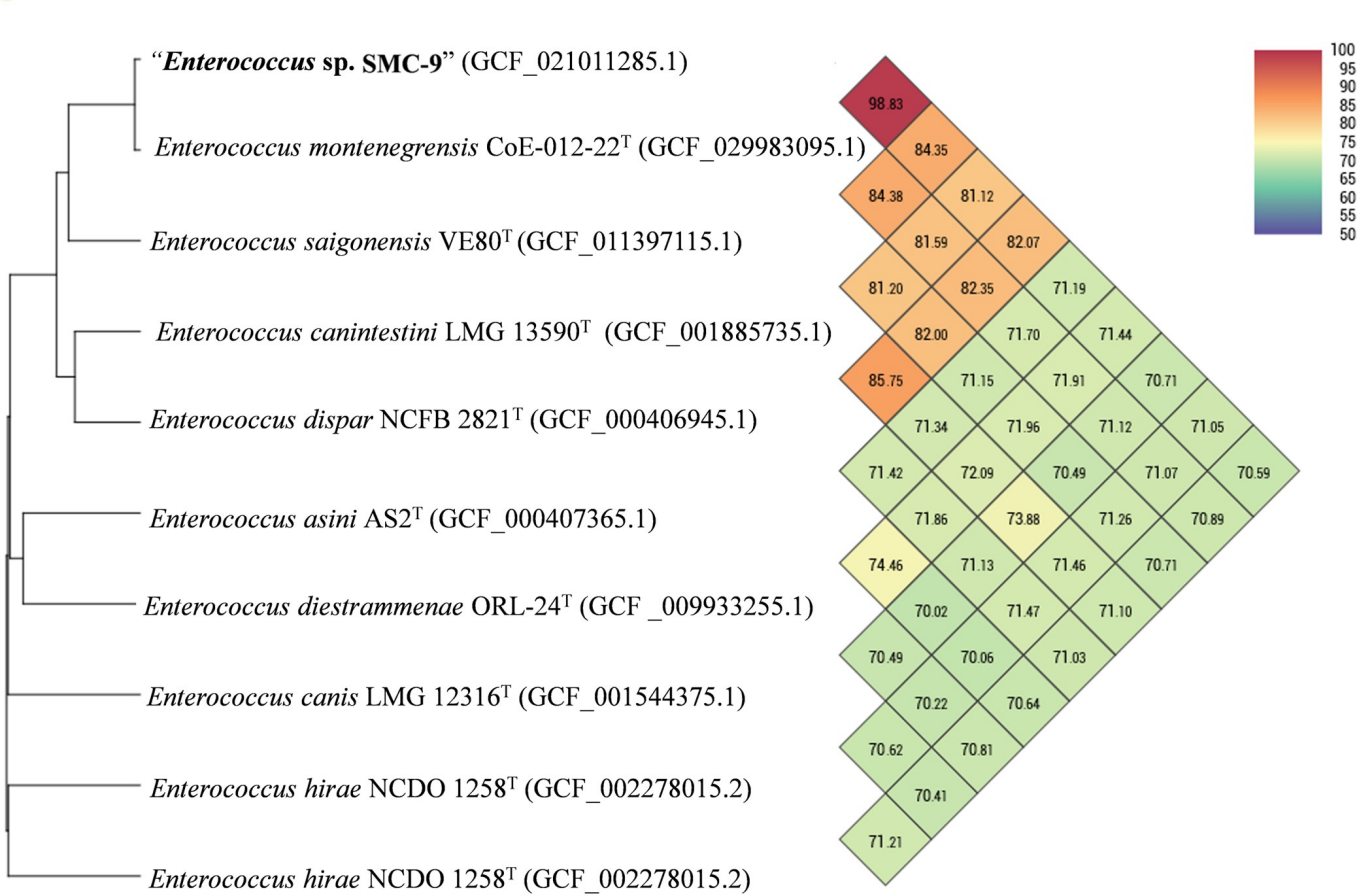
Heatmap generated with Ortho ANI values calculated using the OAT software for strain SMC-9 and related strains within the genus *Enterococcus*.

In the phylogenomic tree based on genome sequences (Fig 3), strain SMC-9 formed a distinct clade with *E*. *montenegrensis* CoE-012-22^T^, *E*. *canintestini* LMC 13590^T^, *E*. *saigonensis* VE80^T^, and *E*. *dispar* NCFB 2821^T^. Notably, the closest clustering was observed with *E*. *montenegrensis* CoE-012-22^T^. These findings are consistent with those observed in the phylogenetic tree based on 16S rRNA gene sequences. The ANI and isDDH values between strain SMC-9 and related strains of the genus *Enterococcus* were 70.4–98.7% and 20.2–90.9%, respectively. The highest ANI and isDDH values (98.7% and 90.9%, respectively) were observed between strain SMC-9 and *E*. *montenegrensis* CoE-012-22^T^ (Table 2 and Fig 4), exceeding the thresholds for the delineation of prokaryotic species (95–96% for ANI and 70% for isDDH) [35]. Based on the results of phylogenetic and phylogenomic analyses, we suggest that strain SMC-9 belongs to the same species as *E*. *montenegrensis* CoE-012-22^T^.

**Table 2:** Average nucleotide identity (ANI) and *in silico* DNA-DNA hybridization (isDDH) values between strain SMC-9 and related strains of the genus *Enterococcus*.

Species	Strain	RefSeq accession no.	ANI (%)	isDDH (%)
*Enterococcus montenegrensis*	CoE-012-22^T^	GCF_029983095.1	98.7	90.9
*Enterococcus saigonensis*	VE80^T^	GCF_011397115.1	84.3	27.9
*Enterococcus dispar*	ATCC 51266^T^	GCF_000406945.1	81.9	25.4
*Enterococcus canintestini*	DSM 21207^T^	GCF_001885735.1	81.0	24.4
*Enterococcus hirae*	ATCC 9790^T^	GCF_000271405.2	72.1	25.5
*Enterococcus asini*	ATCC 700915^T^	GCF_000407365.1	71.8	23.7
*Enterococcus diestrammenae*	JM9A	GCF_009933255.1	71.8	22.1
*Enterococcus italicus*	DSM 15952^T^	GCF_001885995.1	71.7	22.9
*Enterococcus canis*	DSM 17029^T^	GCF_001885805.1	71.6	22.8
*Enterococcus innesii*	DB-1	GCF_022846515.1	71.5	26.4
*Enterococcus lactis*	CX 2–6_2	GCF_019343125.1	71.5	24.3
*Enterococcus durans*	BDGP3	GCF_002277935.1	71.4	24.3
*Enterococcus gilvus*	ATCC BAA-350^T^	GCF_000407545.1	71.1	23.8
*Enterococcus hermanniensis*	DSM 17122^T^	GCF_001885945.1	71.1	20.2
*Enterococcus malodoratus*	NCTC 12365^T^	GCF_900447955.1	70.8	25.3
*Enterococcus pallens*	ATCC BAA-351^T^	GCF_000393975.1	70.7	23.7
*Enterococcus florum*	Gos25-1^T^	GCF_004309355.1	70.4	23.6

**Fig 5:**
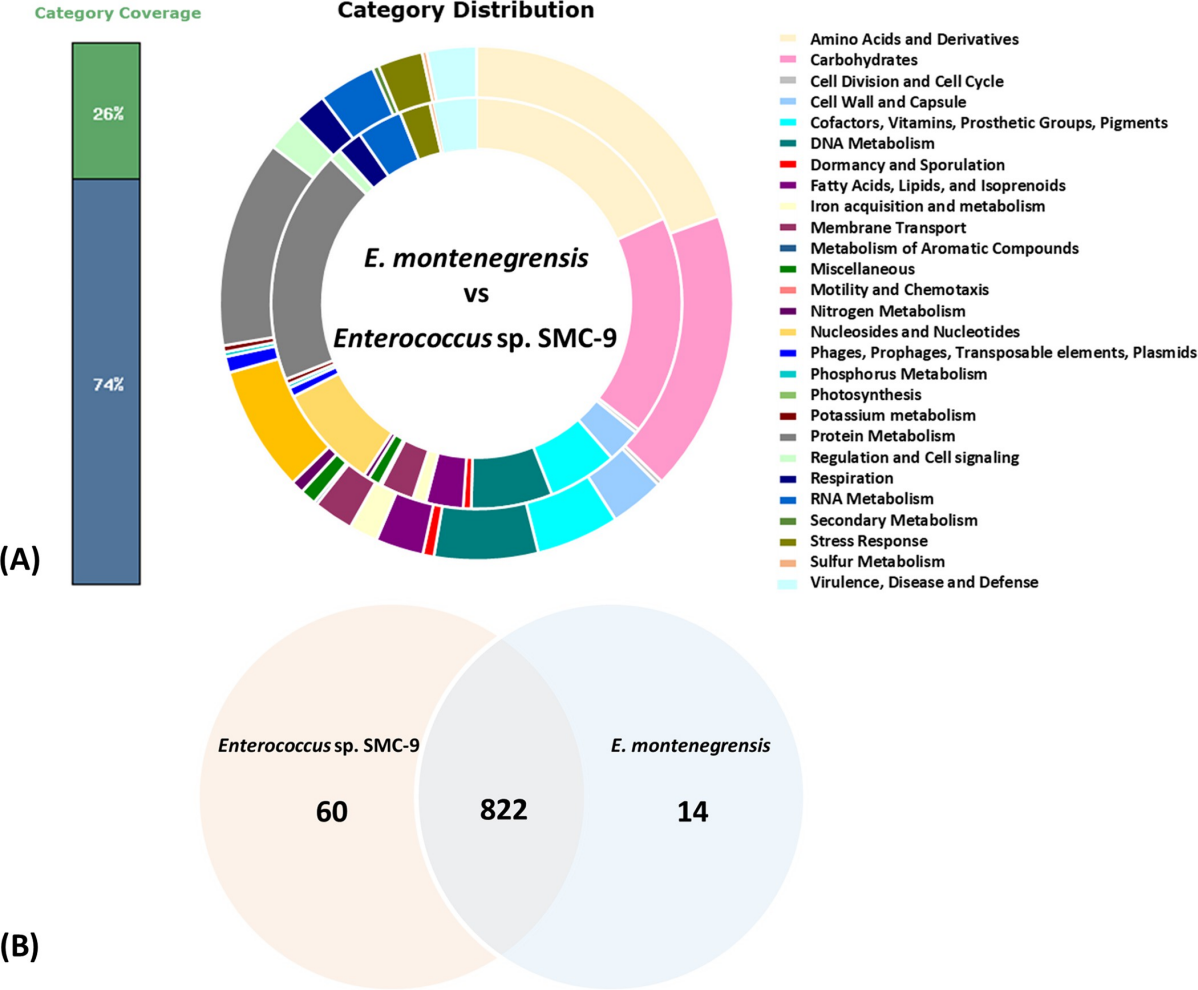
Genome comparison between *E*. *montenegrensis* CoE-012-22^T^ and *Enterococcus* sp. SMC-9. (A) Compositions of gene ontology categories with the inner ring representing the genome of *E*. *montenegrensis* CoE-012-22^T^ and the outer ring representing the genome of strain SMC-9, (B) Venn diagram showing the number of shared and specific gene orthologs within the genomes.

While strain SMC-9 and strain CoE-012-22^T^ share highly similar genomes, differences were noted in their genomic profiles. Comparative genomic analysis on the RAST server, focusing on the chromosomal regions that encode gene functions, revealed a high degree of similarity between the two strains, with 822 matching categories. However, strain SMC-9 exhibited 60 unique gene categories not found in *E*. *montenegrensis* CoE-012-22^T^, while *E*. *montenegrensis* CoE-012-22^T^ had 14 unique gene categories not present in strain SMC-9 (Fig 5). The dominant functional gene categories, including those involved in Amino Acids and Derivatives, Carbohydrates, and Protein Metabolism, were mostly shared between strain SMC-9 and *E*. *montenegrensis* CoE-012-22^T^. However, strain SMC-9 exhibited 20 additional genes in the Amino Acids and Derivatives category and 11 additional genes in the Carbohydrates category compared to *E*. *montenegrensis* CoE-012-22^T^. Of particular interest is the Cell Wall and Capsule category, common to both strains. Within this category, genes responsible for “Exopolysaccharide Biosynthesis” were exclusively found in strain SMC-9 (S1 Table). These functional genes play a crucial role in biofilm formation, which provides a protective environment for bacteria, enhancing their survival against adverse conditions, including immune responses and antibiotic treatments [36]. Although the VirulenceFinder analysis did not identify any specific virulence genes in strain SMC-9, the KEGG analysis revealed that this strain uniquely harbors four genes involved in the L-rhamnose biosynthesis pathway (*rmlA*, *rmlB*, *rmlC*, and *rmlD*). Rhamnose-rich cell wall polysaccharides have been reported to affect virulence and colonization potential in the host gut [37, 38]. Thus, the presence of these genes in strain SMC-9 may have facilitated its colonization of the biliary tract and contributed to the development of cholangitis in the patient.

The KEGG analysis detected no genes related to primary or secondary bile salt metabolism in strain SMC-9 and *E*. *montenegrensis* CoE-012-22^T^. However, it revealed that both strains shared *xthA* and *nfo*, which are required for base excision repair to overcome DNA damage caused by bile salts. They also possessed *dinB* and *recA*, which are necessary for SOS-associated DNA repair for bile-induced DNA damage [39, 40]. Additionally, they shared *mrcA*, a gene encoding penicillin-binding protein 1A, which is known to be related to bile tolerance [39, 41]. These findings suggest that both strains are tolerant to bile, as demonstrated by their growth on bile esculin agar.

Using PathogenFinder, strain SMC-9 was predicted to be a human pathogen with a probability of 0.897 and matched to 10 pathogenic protein families: 1 SSU ribosomal protein S19P, 1 30S ribosomal protein S21, 1 polyribonucleotide nucleotidyltransferase, 1 ribosomal protein L29, 2 conserved hypothetical proteins from *Streptococcus mitis*, 1 conserved hypothetical protein from *Staphylococcus aureus*, 1 conserved hypothetical protein from *Enterococcus faecalis*, and 2 hypothetical proteins from *Staphylococcus aureus*. In contrast, PathogenFinder analysis predicted *E*. *montenegrensis* CoE-012-22^T^ as non-pathogenic [7]. These findings indicate that strain SMC-9 is a causative agent of cholangitis and possesses pathogenic traits distinct from *E*. *montenegrensis* CoE-012-22^T^.

### Antimicrobial susceptibility testing results

The AST results obtained using VITEK 2 were as follows: ampicillin, ≤2 μg/mL (susceptible); ciprofloxacin, 1 μg/mL (susceptible); erythromycin, 4 μg/mL (intermediate); levofloxacin, 1 μg/mL (susceptible); linezolid, 2 μg/mL (susceptible); nitrofurantoin, ≤16 μg/mL (susceptible); norfloxacin, 4 μg/mL (susceptible); penicillin, 0.5 μg/mL (susceptible); Quinupristin-dalfopristin, 2 μg/mL (intermediate); teicoplanin, ≤0.5 μg/mL (susceptible); tetracycline, >16 μg/mL (resistant); vancomycin, ≤0.5 μg/mL (susceptible); high-level gentamicin (susceptible); high-level streptomycin (susceptible). While certain species within the genus *Enterococcus*, particularly common hospital isolates such as *E*. *faecium* and *E*. *faecalis*, are known to exhibit resistance to various antibiotics [42, 43], strain SMC-9, albeit isolated in hospital setting, showed susceptibility to most antibiotics tested. This susceptibility profile holds particular significance given the frequent occurrence of antibiotic-resistant *Enterococcus* strains in hospital environments.

The genome analysis using the CARD and ResFinder revealed that the tetracycline resistance gene *tet*(M) was present in the genome of strain SMC-9, which accounts for the high tetracycline MIC value of this strain (>16 μg/mL). In enterococci, *tet*(M), which encodes a ribosomal protection protein, is the most frequently encountered tetracycline resistance gene [44, 45]. In contrast, *E*. *montenegrensis* CoE-012-22^T^ lacked *tet*(M), explaining its susceptibility to tetracycline [7]. The CARD also identified *vanY* in the *vanB* cluster (*vanY*_*B*_) and *vanT* in the *vanG* cluster (*vanT*_*G*_) in the genomes of both strain SMC-9 and *E*. *montenegrensis* CoE-012-22^T^. *vanY*_*B*_ encodes a D,D-carboxypeptidase that cleaves the terminal D-Ala from peptidoglycan precursors, preventing the binding of vancomycin, while *vanT*_*G*_ encodes a membrane-bound serine racemase, which converts L-Serine to D-Serine, the key substrate for the D-Ala-D-Ser-based vancomycin resistance mechanism [46]. Despite the presence of *vanY*_*B*_ and *vanT*_*G*_, both strain SMC-9 and *E*. *montenegrensis* CoE-012-22^T^ lacked the complete gene cassettes required for vancomycin resistance, rendering these strains vancomycin-susceptible.

In conclusion, strain SMC-9, isolated from bile of a patient with acute cholangitis, demonstrated key differences in virulence and antibiotic resistance gene profiles compared to *E*. *montenegrensis* CoE-012-22^T^, which was isolated from dried beef sausage, despite both strains belonging to the same species. Notably, genes related to exopolysaccharide biosynthesis and the L-rhamnose biosynthesis pathway were found exclusively in strain SMC-9, suggesting their role in the strain’s colonization of the biliary tract and its involvement in cholangitis. Additionally, the tetracycline resistance gene *tet(M)*, which was absent in *E*. *montenegrensis* CoE-012-22^T^, was identified in strain SMC-9, explaining its high tetracycline MIC (>16 μg/mL). These findings underscore the unique pathogenic traits of strain SMC-9 compared to *E*. *montenegrensis* CoE-012-22^T^. This study highlights significant genetic and phenotypic variations that can exist among strains classified within the same species, emphasizing the critical need for strain typing to evaluate their potential impact on patient outcomes and public health. In the context of increasing awareness of antibiotic-resistant *Enterococcus* infections, continued research into the characteristics of variant strains isolated from patients, particularly those with multidrug resistance, is of critical clinical importance.

### Description of *Enterococcus* sp. strain SMC-9

Cells are Gram-stain-positive, spherical- or ovoid-shaped cocci with 0.7–1.2 μm in diameter that usually occur in pairs or short chains. When cultured on MRS agar, growth is observed at temperatures between 25 and 37°C, with an optimal temperature of 37°C. The strain can grow in NaCl concentrations ranging from 0% to 5.5%, with optimal growth occurring at 3.0% NaCl. Additionally, it is capable of growing in a pH range from 5.0 to 7.5, with optimal growth observed between pH 6.0 and 6.5. Colonies grown on MRS agar after incubation at 37°C for 48h are circular, convex, entire, smooth, shiny, greyish-white, and 1–2 mm in diameter. The strain is catalase- and oxidase-negative, lacks the Lancefield group D antigen, and is capable of growing on bile esculin agar. The strain is positive for acetoin production, hippuric acid hydrolysis, β-glucosidase hydrolysis, pyrrolidonyl arylamidase, leucine aminopeptidase, and arginine dihydrolase, and negative for α-galactosidase, β-glucuronidase, β-galactosidase, and alkaline phosphatase. Acid is produced from D-ribose, D-lactose, and D-trehalose, but not from L-arabinose, D-mannitol, D-sorbitol, inulin, D-raffinose, starch, and glycogen.

The strain is SMC-9 (= KCTC 21174 = JCM 34907), isolated from bile of a patient with cholangitis. The draft genome of the strain is 2.81 Mb in size with a DNA G+C content of 37.5 mol%.

## Supporting information

S1 Fig Subsystem coverage and category distribution of strain SMC-9 based on the RAST annotation results. (TIF)

S2 Fig Circular genome map of strain SMC-9. (TIF)

S1 Table Comparative genome analysis of strain SMC-9 and *E*. *montenegrensis* CoE-012-22^T^ based on the RAST server. (XLSX)
