# Supplementary figures and images for: Characterization of an Enterococcus sp. SMC-9 strain isolated from bile of a patient with cholangitis
Source: PLoS One. 2024 Dec 2;19(12):e0312953. doi: 10.1371/journal.pone.0312953 (PMC11611144; doi:10.1371/journal.pone.0312953)

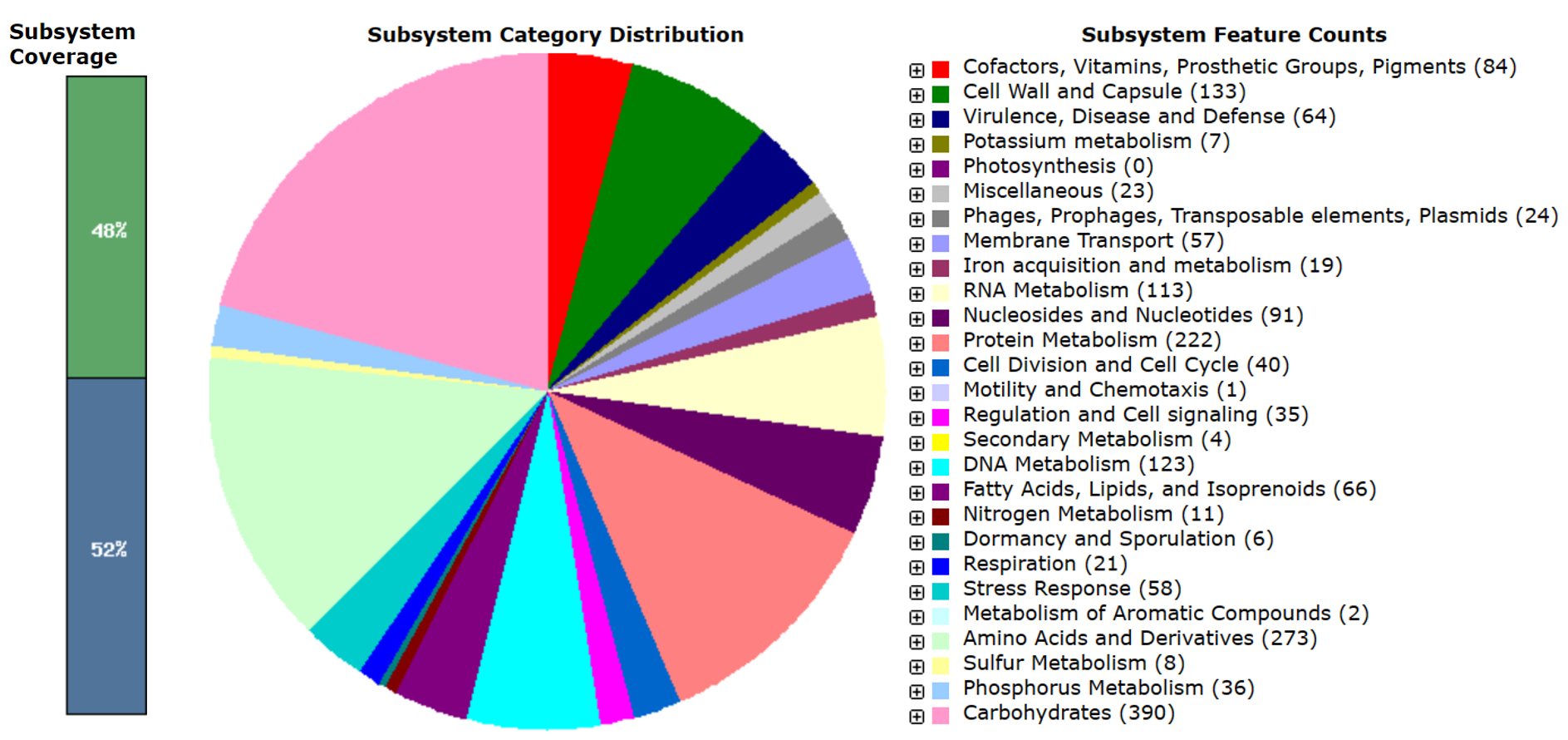

Supplement: S1 Fig — (TIF) [file pone.0312953.s001.tif]

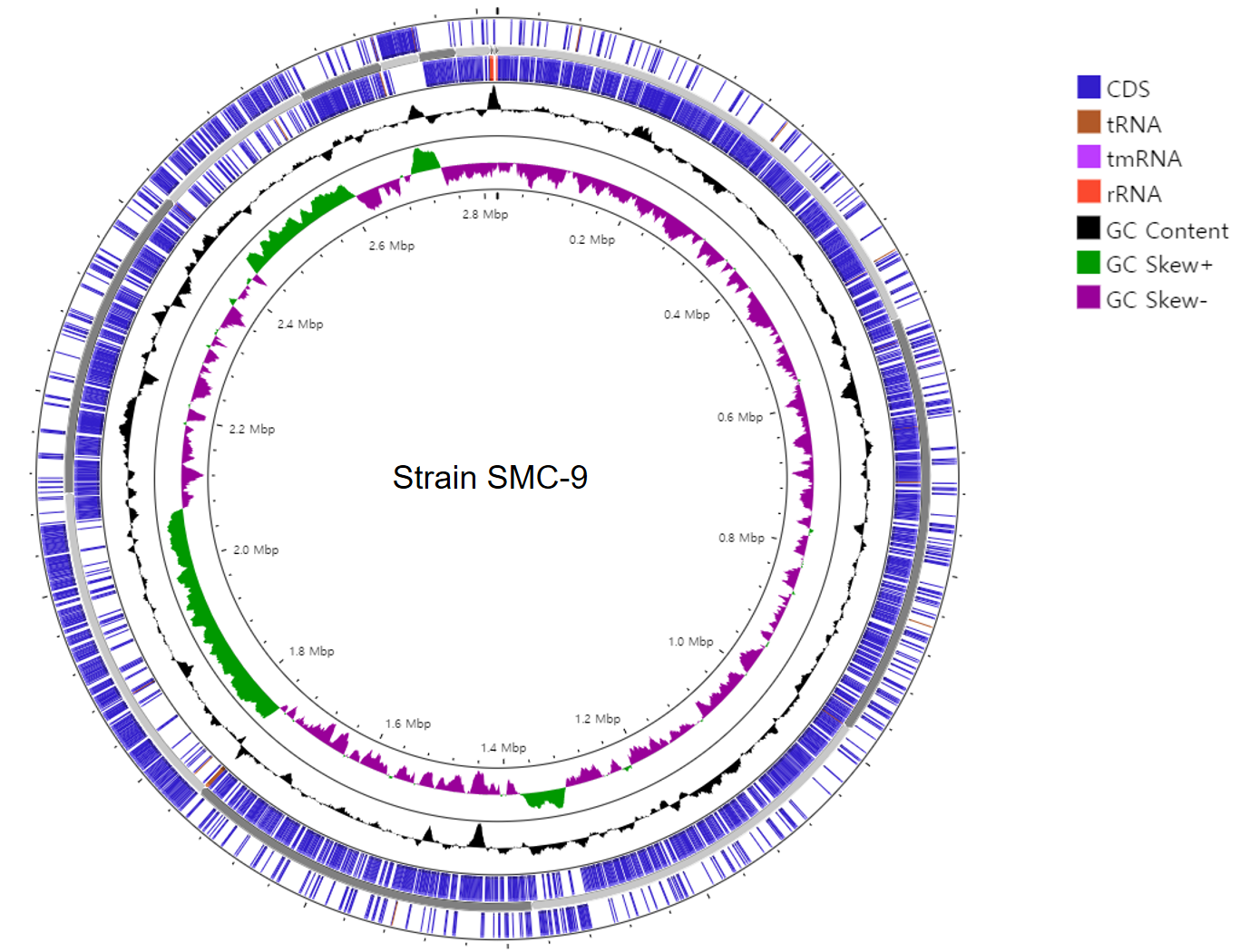

Supplement: S2 Fig — (TIF) [file pone.0312953.s002.tif]
